# Soil Physicochemical Parameters and Bibliographically Inferred Microbial Diversity as Drivers of Early-Stage Biodegradation of *Colocasia esculenta* and *Manihot esculenta* Starch Bioplastics in Three High-Andean Soils of Ecuador

**DOI:** 10.3390/polym18121506

**Published:** 2026-06-16

**Authors:** María Soledad Núñez Moreno, Georgina Esther Carmilema Yungan, María Gabriela Arias Garnica, David Esteban Puyol Guevara

**Affiliations:** Escuela Superior Politécnica de Chimborazo (ESPOCH), Riobamba 060155, Ecuador; soledad.nunez@espoch.edu.ec (M.S.N.M.); georgina.carmilema@espoch.edu.ec (G.E.C.Y.); mariag.arias@espoch.edu.ec (M.G.A.G.)

**Keywords:** starch bioplastics, *Colocasia esculenta*, *Manihot esculenta*, soil biodegradability, gravimetric weight loss, microbial diversity, high-Andean soils, Global South

## Abstract

Single-use plastic residues persist in agricultural and peri-urban soils of the Ecuadorian Andes. Regionally sourced starch-based films are a plausible local replacement for short-lifetime petroleum plastics, yet field-relevant degradation data for tropical high-altitude soils remain scarce. This study evaluated the soil biodegradability of bioplastic films produced from *Colocasia esculenta* (malanga blanca) and *Manihot esculenta* (yuca) across three contrasting soils from Chimborazo, Ecuador (ESPOCH, San Andrés and Río Chimborazo; 2825–3249 m a.s.l.) as a function of their physicochemical properties and bibliographically inferred microbial context. The films were prepared by citric acid starch extraction, glycerol plasticization and carboxymethylcellulose reinforcement; the gravimetric weight loss was tracked on days 0, 11, 18, 27, 40 and 47 on n = 20–21 film replicates per soil × feedstock combination, with the soils characterized by their pH, electrical conductivity and organic matter. After 47 days, the malanga films reached 42.3 ± 13.6%, 22.9 ± 10.7% and 54.1 ± 19.3% mean (±standard deviation, SD) weight loss in the ESPOCH, San Andrés and Río Chimborazo soils, respectively; the yuca films reached 24.4 ± 6.5%, 21.1 ± 6.8% and 49.4 ± 18.7%. The between-soil differences were statistically significant at 47 days according to the analysis of variance (ANOVA) (malanga: F = 22.17, *p* < 0.001; yuca: F = 34.08, *p* < 0.001; Tukey’s Honestly Significant Difference (HSD)), with the results corroborated by the Kruskal–Wallis method (H = 29.16 and 37.05; both *p* < 0.001), given the partial departure from normality identified by the Shapiro–Wilk test. The ordering of degradation departed from the bulk organic matter ranking, indicating that microbial community composition, rather than organic matter quantity alone, was the proximal driver. These findings extend the scarce evidence base on cassava/taro film degradation under high-Andean conditions.

## 1. Introduction

The soils in the Ecuadorian highlands receive a large share of the country’s unmanaged plastic waste; national figures place plastics at roughly 11% of household refuse, which translates into about 531 × 10^3^ t of synthetic plastics generated annually [1]. Because conventional packaging polymers are overwhelmingly manufactured from non-renewable fossil feedstocks—the share commonly quoted for global plastic production exceeds 99% [2]—the waste that reaches Andean soils and watercourses is both persistent and structurally tied to an extractive value chain that small producers cannot redesign from below. The environmental load is therefore compounded by an economic one: the rural Sierra has no meaningful domestic substitute for short-lifetime films, mulches and packaging, and what it produces in surplus—starch-rich tubers such as *Colocasia esculenta* and *Manihot esculenta*—is undervalorized [3]. The motivation for the present study sits precisely on that hinge.

Starch-based bioplastics are the most thoroughly characterized class of bio-based films available to tropical developing regions. Their appeal rests on three well-documented properties: renewability, hydrolytic susceptibility, and direct compatibility with casting protocols at laboratory and pilot scale [4,5]. Amylose and amylopectin provide a glycosidic backbone that amylolytic microorganisms can target enzymatically [6], and the two Andean tubers selected here offer attractive starch yields—30–85% in *Colocasia esculenta* and around 30.85% in *Manihot esculenta* [7,8]—together with a strong regional production base in Ecuador’s coastal and Amazonian provinces. Proof-of-concept casting with citric acid extraction, glycerol plasticization and carboxymethylcellulose reinforcement has also been documented in the regional literature [9], meaning that the study is not trying to establish whether these tubers can yield a film—that is already known.

What remains open, and what matters for translating this chemistry into an environmental argument, is the soil side of the interaction. The rate and extent of starch film mineralization are governed jointly by the film chemistry and by the receiving edaphic environment: the pH, electrical conductivity, organic matter, moisture regime and—most directly—the composition and potential of the soil microbial community [6,10,11]. Recent work has shown that two-phase degradation kinetics are the rule rather than the exception: an early enzymatic hydrolysis phase dominates the accessible amorphous fractions, after which a slower mass transfer-limited phase governs the residual polymer [12,13,14]. This kinetic structure has two consequences for experimental designs. First, the early-stage gravimetric loss precisely captures the phase in which the soil’s microbial and physicochemical heterogeneity exerts its strongest influence, providing a window into the soil–polymer interaction [15,16]. Second, the gravimetric loss is not indicative of complete mineralization; plasticizer leaching and soil adhesion may inflate or obscure the signal if the controls are not handled in the correct way [16,17].

When the existing literature is read with the Global South in mind, the gap sharpens. Quantitative studies of starch film degradation still cluster around temperate compost-amended soils or controlled laboratory media; region-specific, field-relevant data for tropical and high-altitude soils in developing countries are sparse, and life-cycle benchmarking for cassava/taro agricultural films is effectively absent [3,18]. The Andean highlands compound this absence with a specific edaphic signature—alkaline pH, low-to-moderate organic matter, cool mean temperatures and distinct microbial assemblages along altitudinal gradients—that is not interchangeable with lowland tropical or temperate soils [19,20]. Recent comparative work has shown that biodegradation kinetics of starch and starch-blend films vary by an order of magnitude across soils with different microbial structures and physicochemical settings, with the rate-limiting step shifting from enzymatic attack to mass transfer constraints as a system departs from the optimum [12,13,21]. The existing work that has reported on cassava or taro degradation in tropical conditions has typically done so at single sites, without contrasting soils, and without an explicit treatment of how the microbial community composition modulates the early phase. The empirical record on whether Andean soils with different physicochemical and microbiological characters degrade these films at meaningfully different rates remains, at present, incomplete.

Two further points bear on how the gap is framed here. First, microbial community composition cannot be taken as a proxy for the bulk organic matter content: several recent syntheses have reported that the organic matter quantity and moisture are themselves co-correlated within single-soil landscapes, which limits any attempt to attribute the signal to organic matter alone [17,22]. Any study that wishes to make a microbial argument therefore needs both a physicochemical characterization and, at least, a defensible reconstruction of the microbial assemblage. Second, incomplete degradation at early time points is not an innocuous outcome: residual fragments of starch films can themselves enter the bio-microplastic stream when environmental conditions are sub-optimal [23]. Any empirical claim that a starch-based film is “environmentally compatible” has to be constrained by this possibility rather than asserted against it.

Against this background, the study evaluates the soil biodegradability of bioplastic films produced from *Colocasia esculenta* and *Manihot esculenta* in three Andean soils with contrasting physicochemical and microbiological characters—ESPOCH, San Andrés and the margins of the Río Chimborazo in San Juan parish (2825–3249 m a.s.l.)—over a 47-day window at six sampling times. The work is designed as an exploratory–comparative early-stage investigation: its endpoint is a quantified contrast between feedstocks and among soils, interpreted through the published mechanistic framework of starch mineralization, rather than a predictive kinetic model of complete mineralization, for reasons that are made explicit in the Methods section. The research question is therefore precise: how do the physicochemical properties and microbial diversity of soil influence the biodegradability of bioplastics produced from *Colocasia esculenta* and *Manihot esculenta*? The associated hypothesis is that differences in the physicochemical properties and microbial diversity significantly influence the degradation rate, with higher rates expected in soils with greater organic matter content and microbial diversity. The general objective is to evaluate the soil biodegradability of the two starch-based films as a function of the physicochemical and microbiological properties of three contrasting Andean soils, producing region-specific evidence for a polymer class for which field data in the high-Andean Global South have so far been absent.

## 2. Materials and Methods

### 2.1. Study Scope and Analytical Framing

This work was designed as an exploratory–comparative evaluation of early-stage soil biodegradation of two starch-based bioplastic films under three contrasting Andean edaphic environments. Its endpoint was a quantified contrast between feedstocks and among soils, read against a mechanistic framework of two-phase starch mineralization [6,12,13] rather than a predictive kinetic model of complete mineralization. The choice was deliberate, and three analytical consequences are declared here so that Section 2, Section 3 and Section 4 read consistently.

First, the experiment was carried out over 47 days with gravimetric weight loss as the primary response. Gravimetric weight loss is a defensible proxy for the early phase of soil biodegradation of starch-based films, provided that environmental conditions are controlled and measurements are standardized [15,16]; it is not, however, equivalent to complete mineralization, because early weight loss partly conflates biotic hydrolysis with plasticizer release [17]. Second, the microcosms were not archived with film-level raw time series per replicate; this limited the inferences to aggregated gravimetric responses across predefined sampling times, and ruled out a meaningful fit of Linear Mixed Models (LMMs), non-linear Boltzmann/Hill kinetics, or principal component analysis (PCA) over the soil × film matrix [24,25]. This was a documentary constraint, not a methodological preference, and it frames the statistical section below. Third, the original thesis design placed multiple films inside shared containers—a pseudoreplicated configuration, per [24]—which is declared and handled transparently in Section 2.6 and revisited in Section 4.

### 2.2. Study Site and Experimental Setting

**Figure 1:**
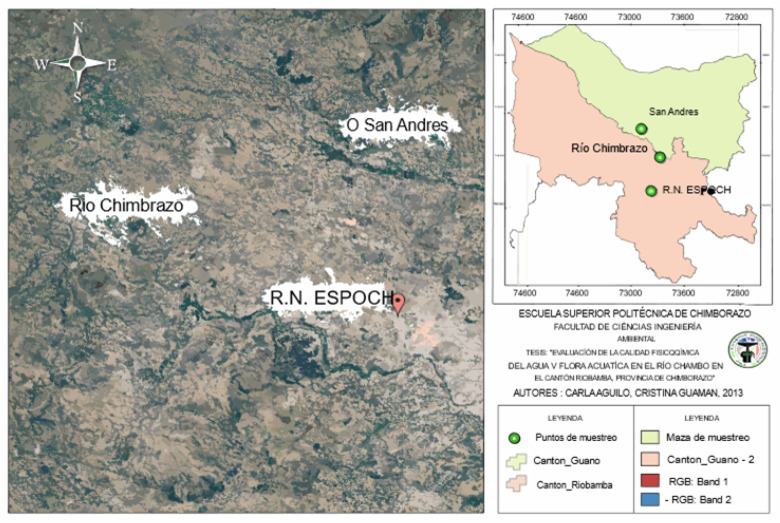
Geographical setting and sampling scheme of the three Andean soils analyzed in this study. Note: The map identifies the three sampling locations in the province of Chimborazo—ESPOCH campus (S1); San Andrés (S2); and Río Chimborazo, San Juan parish (S3)—together with the UTM coordinates, elevation, and a schematic of the soil × film burial configuration. Source of inset map adapted from Aguilo and Guaman (2013).

The laboratory work was performed at the Laboratorio de Procesos Industriales and at the GIDAC research laboratory (Facultad de Ciencias, Escuela Superior Politécnica de Chimborazo, ESPOCH), with the burial phase conducted at the greenhouse facilities of the Facultad de Recursos Naturales of the same institution, in Riobamba, province of Chimborazo, Ecuador (Figure 1). Three soils were sampled along an altitudinal gradient spanning 2825–3249 m a.s.l. within the Chimborazo highlands, selected a priori for their edaphic and bibliographically documented microbiological contrasts rather than for proximity or convenience (Section 2.5).

### 2.3. Starch Extraction from Colocasia esculenta and Manihot esculenta

Raw tubers of *Colocasia esculenta* (malanga blanca) and *Manihot esculenta* (yuca) were washed, peeled, and cut into cubes of 5–6 cm^3^ following Karnwal et al. (2025) [9], which consolidated the procedure originally described by Graf et al. (2025) [26]. The cubes were submerged in a 3% (*w*/*v*) citric acid solution and ground until a homogeneous slurry was obtained. The slurry was filtered through nylon mesh to remove the fibers, and the filtrate was allowed to decant for 4 h at 4 °C [7]; the supernatant was discarded, and the sedimented starch was rinsed three times with distilled water, with centrifugation during the final rinse to recover the residual starch from the supernatant. The recovered starch was oven-dried at 55 °C for 24 h (ESCO OFA-110-8), milled (Corona grain mill), and sieved into a homogeneous fine powder [7]. The dried starch was stored in sealed plastic containers until film casting. This method is reproducible and has been repeatedly documented for malanga and yuca in the regional literature [7,9].

All reagents used in the film preparation—citric acid (analytical grade, ≥99%), glycerol (analytical grade, ≥99.5%), carboxymethylcellulose sodium salt (medium viscosity, food grade), and acetic acid (analytical grade, ≥99.7%)—were obtained from a certified scientific distributor in Ecuador, with the analytical-grade purity certificates retained by the authors. An OHAUS AX224 analytical balance (OHAUS Corporation, Parsippany, NJ, USA) was used for the gravimetric measurements with a readability of 0.0001 g. A universal testing machine (manufacturer details available from the authors on request) was used for tensile testing. Daily ambient monitoring of soil moisture and temperature inside the microcosms was performed with digital thermo-hygrometers.

### 2.4. Bioplastic Film Formulation and Casting

The films were produced using the formulation proposed by Karnwal et al. (2025) [9]. For each batch, 2.5 g of starch was dispersed in 40 mL of distilled water and supplemented with 1 g of NaCl (transparency enhancer and mild antimicrobial agent) and 1 mL of sunflower oil (mold-release agent). To adjust the mechanical performance, 0.75 g of carboxymethylcellulose (CMC) was incorporated as an extender, 6 mL of glycerol (C_3_H_8_O_3_) as a plasticizer, and 2.50 mL of acetic acid (CH_3_COOH) as a chemical modifier intended to reduce the hydrophilic character of starch at the surface [9]; the latter corresponds to approximately 5% (*v*/*v*) of the total liquid volume of the dispersion. The dispersion was homogenized under continuous magnetic stirring at approximately 400 rpm with gradual heating from room temperature to 75 ± 2 °C, holding this temperature for 15 min until the mixture became homogeneous and translucent. The resulting viscous matrix was then poured in fixed aliquots of 20 mL onto aluminum trays of standardized area and dried at 50 °C for 24 h in a convection oven (ESCO OFA-110-8) to obtain continuous films of ≈0.10–0.13 mm nominal thickness [8], which was verified post-drying with a digital micrometer (precision ± 0.01 mm); the replicates outside this thickness window were discarded prior to the burial assay. The films were stored under ambient conditions (22–24 °C, 40–55% relative humidity) until characterization and burial. The glycerol–CMC–acetic acid formulation reproduces a configuration already characterized in the regional literature [9], which is acknowledged as a replicative choice: the contribution of this study is not the formulation itself but its behavior under three contrasting Andean edaphic environments.

### 2.5. Physical–Mechanical Characterization of Bioplastic Films

Three samples per feedstock were characterized for their thickness, transparency, density, Shore A hardness, and tensile/elastic behavior, following the procedural consolidation of Karnwal et al. (2025) [9], with the source protocols indicated below. The thickness was measured with a digital caliper at three points on each square and averaged [27]. The transparency was assigned a categorical rating (transparent/semi-opaque/opaque) by visual panel against a standard grid [28]. The apparent density was calculated as d = m/v, weighing each 3 × 3 cm square on an analytical balance (OHAUS AX224) and estimating the volume from the thickness and area, following Billings et al. (2025) [29]. The Shore A hardness was measured with a Shore A durometer on three distinct points of each 1 × 1 cm subsample [2]. The elastic behavior (reported here as elastic modulus in MPa) was assessed on a universal testing machine (MUE) following Guo et al. (2013) [30]. Reporting of units follows the IUPAC-aligned convention adopted by Polymers (SI units; thickness in mm).

### 2.6. Soil Sampling, Physicochemical Characterization and Microbiological Context

Three composite soil samples were collected from the upper 30 cm of the mineral horizon at the three previously identified locations (Section 2.2) using a stainless-steel cylinder of 100 cm^3^ for the bulk density and moisture determinations and Ziploc bags for the bulk subsamples. The coordinates were recorded with a handheld GPS; the samples were transported on the same day to the Grupo de Investigación en Desarrollo Ambiental y Comunitario (Research Group in Environmental and Community Development, GIDAC) laboratory of the ESPOCH.

The soil pH was determined in a 1:2 (*w*/*v*) soil-to-deionized-water suspension, agitated for 10 min and read after 5 min of settling with a pH meter previously calibrated against buffer solutions with pH 4, 7 and 10 [31]. The electrical conductivity (EC, µS·cm^−1^) was determined on the same supernatant with a conductivity meter. The bulk density and gravimetric moisture (cylinder method) were computed from the wet and oven-dried mass (105 °C, 24 h) of undisturbed cores. The organic matter (OM) content was determined by loss on ignition following a calcination step in a muffle furnace at 360 °C for 2 h on pre-tared crucibles [31], and computed as

%OM = [(P_dry − P_calc)/P_dry] × 100,

where P_dry is the mass of oven-dried soil after removal of the tared crucible, and P_calc is the mass of the same soil after calcination.

The microbiological characterization of the three soils was not performed by direct 16S/ITS sequencing or by microbial biomass quantification on the current cores. Instead, each soil was assigned a microbial diversity context based on prior dedicated studies of these same locations: [19], whose work on microbial diversity in the microcatchment of the Río Chimborazo reports a rich multi-phyla community in the San Juan margin; and [20], who reports the Gram-positive and Gram-negative bacterial loads characteristic of the San Andrés and ESPOCH soils. This constitutes a bibliographically inferred microbiological context, not a direct measurement of the cores incubated in the experiment. This point is declared here, is revisited in the Discussion, and motivates the conservative framing of any microbially mediated interpretation of the results.

### 2.7. Biodegradation Assay (Burial Test)

The burial test followed the gravimetric protocol of Syranidou et al. (2024) [32], which is the procedure upon which the regional literature on starch-based films has converged [33,34]. For each soil × feedstock combination, the films were cut into 3 × 3 cm squares on an analytical balance (OHAUS AX224). The squares were buried at a depth of ≈3 cm in open-top containers mounted within foam (Flex) trays, preserving aerobic conditions at ambient temperature, and incubated for 47 days over the months of November to January. The soil temperature and air humidity in the immediate environment of each tray were logged once per day at the same hour using a digital thermohydrometer (BOE 327), and the paired daily series were retained for use in the moisture–degradation and temperature–degradation descriptive analyses in Section 3.

Blank (control) films of each feedstock were incubated under ambient conditions without soil, as an internal reference for the mass change arising from non-edaphic processes (ambient humidity, handling, and plasticizer volatilization). The gravimetric weight loss was measured at six sampling times (*t* = 0, 11, 18, 27, 40, 47 d). At each sampling, the films were recovered, gently brushed to remove adhered soil particles, and reweighed on the same analytical balance. The percentage weight loss per square was computed as

**Figure 2:**
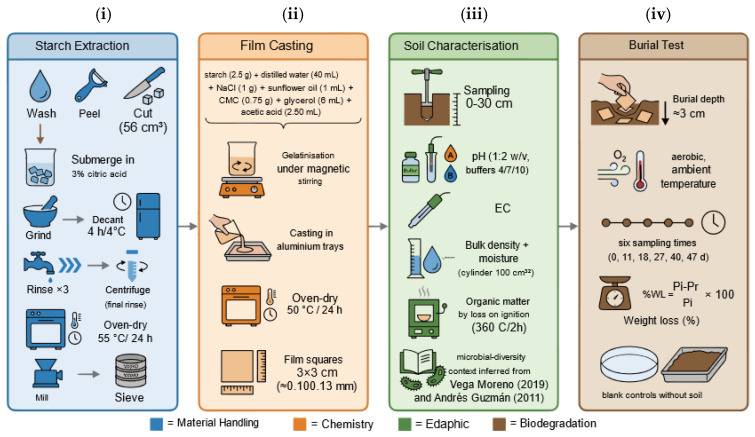
Process flow of experimental pipeline, from starch extraction to gravimetric follow-up. Four stages are shown sequentially: (**i**) raw material preparation and citric acid extraction; (**ii**) film casting with glycerol, CMC and acetic acid; (**iii**) soil sampling, physicochemical characterization and microbiological contextualization; and (**iv**) burial test; gravimetric sampling at t = 0, 11, 18, 27, 40, 47 d; and blank control incubation. Microbial-diversity contextualization based on Vega Moreno (2019) and Andrés Guzmán (2011).

%WL = [(P_i − P_f)/P_i] × 100,

where P_i is the initial mass of the square before burial and P_f is its mass at the given sampling time. Residual soil adhesion remains a recognized source of gravimetric bias in starch film burial assays [16]; the cleaning procedure is reported here at the level of detail available in the experimental notebooks (Figure 2), and the implications of residual adhesion for the interpretation of early-phase data are taken up in Section 4.

### 2.8. Experimental Design Transparency and Handling of Pseudoreplication

Multiple film squares per soil × feedstock combination were incubated in shared containers rather than in fully independent microcosms. Per [24], this configuration is pseudoreplicated: the true experimental unit is the container, not the film. Three implications of this are acknowledged here without evasion.

First, the inferential statistics in Section 2.9 treat the F-statistics and *p*-values from the original one-way ANOVAs as descriptive summaries of the contrast under a nested grouping, not as the outputs of an unconstrained factorial design with independent biological replicates. Second, the nominal degrees of freedom of the within-soil tests are inflated relative to the true design; this is consistent with the conservative scope declared in Section 2.1 and is flagged again in Section 4. Third, the reconstruction proposed by [24]—one independent microcosm per film, with the soil as a fixed block and the time as a repeated-measures factor, fitted by LMMs with the microcosm nested within soil—cannot be executed post hoc on data that were archived as aggregates. It is therefore reported here as the recommended redesign for any follow-up study, not as the present analysis.

### 2.9. Statistical Analysis

Analyses were carried out in Microsoft Excel (descriptive statistics) and in SPSS Statistics v.25 (inferential tests); results were cross-checked across both tools. Physical–mechanical properties are reported as mean ± standard deviation with 95% confidence intervals (CIs), along with minima and maxima (n = 3 films per feedstock). Soil physicochemical properties are reported as point measurements per soil (n = 1 composite sample per site for pH, EC, OM and bulk density, consistent with exploratory–comparative scope explained in Section 2.1).

The weight loss responses were analyzed at two levels, following the consolidation recommended by [35,36]:

**Within-soil contrasts:** For each feedstock, a one-way ANOVA was applied across the sampling times within each soil (α = 0.05) to test whether weight loss progressed significantly during the 47-day window.
**Between-soil contrasts:** For each feedstock, a one-way ANOVA was applied across the soils at the endpoint of the assay to test whether the soils differed in their final weight loss response.

Assumption testing and non-parametric verification: The normality assumption of the one-way ANOVA was assessed by a Shapiro–Wilk test on each soil × feedstock × sampling time group; 13 of the 24 non-baseline groups departed from normality (W = 0.61–0.98, *p* < 0.05). Homogeneity of variances across soils was assessed by Levene’s test, which detected significant heteroscedasticity for the yuca panel at all sampling times (F = 7.7–8.2, *p* < 0.01) and for the malanga panel on day 18 only. Given these departures, the between-soil endpoint contrasts were corroborated with a non-parametric Kruskal–Wallis test, which reproduced the ordering observed in the parametric analysis (H = 29.16 for malanga and H = 37.05 for yuca; both *p* < 0.001). The effect sizes are reported as eta squared (η^2^) for completeness, and post hoc pairwise comparisons among the soils were carried out using Tukey’s HSD with the original α = 0.05. The full output of these tests is provided in Supplementary Material S1.

The descriptive correlations between the edaphic covariates (daily soil proximal humidity, daily air temperature) and weight loss are reported as coefficients of determination (R^2^) per soil × feedstock combination to preserve comparability with the original analysis while avoiding any implicit causal attribution [17]. Given the documented co-correlation between soil moisture and organic matter in small soil panels [17,22], these R^2^ values are presented as descriptive covariation summaries, not as partitioning of causal influence.

The analyses deliberately do not include LMMs, non-linear Boltzmann/Hill kinetic fitting, DT_50_ extraction, PCA of soil variables, or PERMANOVA. These procedures are the appropriate Q1 toolkit for a study with microcosm-level raw time series and direct metagenomic characterization of the inoculum [6,14,24]; they are recommended in Section 5 for the next iteration of this work. The present work does not claim them and does not substitute for them with extrapolations from the aggregate data, in line with the evidentiary caution advocated by [25] for DT_50_-type metrics derived outside their validated support.

### 2.10. Environmental Impact Matrix

In parallel with the biodegradation assay, a qualitative environmental impact assessment was applied to the main activities of the bioplastic production pathway (raw material collection, starch extraction, film casting, and burial test). The impacts were scored for their nature, magnitude and extent under the qualitative–quantitative scheme described by [37] and subsequently consolidated into a compatibility matrix. This matrix is reported and discussed in Section 3, but it is not used as a substitute for a full life-cycle assessment; a region-specific LCA for cassava/taro agricultural films remains an open gap in the Global South [3,18].

## 3. Results

The results are organized along the four thematic axes mapped in the Coherence Blueprint: the material effect (physical–mechanical characterization of the two films); the soil context (edaphic and microbiological characterization of the three locations); the temporal biodegradation response (within- and between-soil contrasts plus the soil-free control baseline); and the environmental covariates (humidity- and temperature-related descriptive relationships) and the qualitative environmental impact of the production pathway. Their interpretation is deferred to Section 4.

### 3.1. Physical–Mechanical Characterization of the Bioplastic Films

The consolidated descriptive statistics of the films produced from *Colocasia esculenta* and *Manihot esculenta* are summarized in Table 1. The *Colocasia esculenta* films showed a lower mean density (0.369 ± 0.039 g·mL^−1^; range 0.32–0.40; 95% CI 0.271–0.467) than the *Manihot esculenta* films (0.574 ± 0.096 g·mL^−1^; range 0.47–0.66; 95% CI 0.335–0.814). The Shore A hardness averaged 10 ± 1 (range 9–11; 95% CI 7.516–12.484) for the malanga films and 13.3 ± 0.577 (range 13–14; 95% CI 11.899–14.768) for the yuca films. The elastic modulus was 2.240 ± 0.165 MPa for the malanga (range 2.07–2.40; 95% CI 1.829–2.650) and 1.887 ± 0.047 MPa for the yuca (range 1.85–1.94; 95% CI 1.769–2.004). The thickness averaged 0.10 ± 0.015 mm for the malanga (range 0.09–0.12 mm; 95% CI 0.065–0.141) and 0.13 ± 0.020 mm for the yuca (range 0.11–0.15 mm; 95% CI 0.080–0.179). The transparency was categorical: the three malanga replicates (A1–A3) were classified as semi-opaque and the three yuca replicates (B1–B3) as opaque.

### 3.2. Physicochemical and Microbiological Characterization of the Three Soils

The three soils were alkaline with low-to-moderate electrical conductivity (Table 2). San Andrés showed the highest pH (8.36) and the highest EC (130.7 µS·cm^−1^), followed by Río Chimborazo (pH 8.17; EC 108.7 µS·cm^−1^) and ESPOCH (pH 8.12; EC 114.8 µS·cm^−1^). The grand means were 8.22 (pH) and 118.1 µS·cm^−1^ (EC). The organic matter content was ranked ESPOCH (2.3%) > Río Chimborazo (1.9%) > San Andrés (0.9%). The gravimetric moisture in the 100 cm^3^ cylinders followed a different ordering, with ESPOCH the wettest (23.84%), followed by San Andrés (14.69%) and Río Chimborazo (13.91%). The apparent bulk densities were 1.58 g·cm^−3^ (ESPOCH), 1.47 g·cm^−3^ (San Andrés) and 1.61 g·cm^−3^ (Río Chimborazo).

The microbiological context assigned to each soil, as declared in Section 2.6, was drawn from prior regional studies: San Andrés and ESPOCH were characterized by Gram-positive and Gram-negative bacterial assemblages, with a bacillar morphotype predominant in the ESPOCH samples [20]; Río Chimborazo was characterized by a richer spectrum comprising the bacterial phyla *Firmicutes*, Verrucomicrobia, *Cyanobacteria*, *Proteobacteria*, *Actinobacteria* and *Bacteroidetes*, along with the fungal divisions *Ascomycota*, *Basidiomycota*, Mortierellomycota and Gloderomycota [19].

### 3.3. Biodegradation Trajectories and Statistical Contrasts

**Figure 3:**
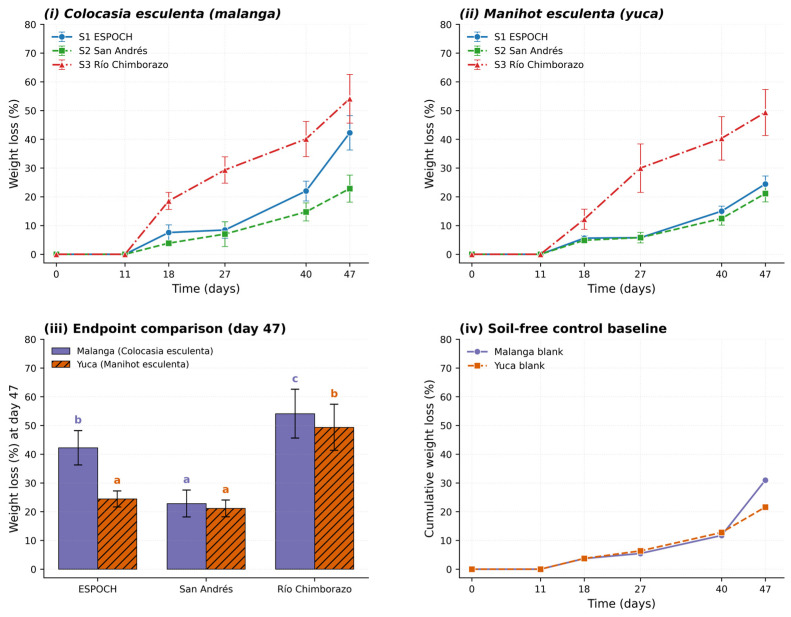
Temporal trajectories of gravimetric weight loss (%) for *Colocasia esculenta* and *Manihot esculenta* bioplastic films over 47 days. (**i**) *Colocasia esculenta* across the three Andean soils—S1 is ESPOCH (blue, circles, solid line), S2 is San Andrés (green, squares, dashed line), and S3 is Río Chimborazo (red, triangles, dot-dashed line); the error bars represent the 95% confidence interval of the mean (n = 20 per time point). (**ii**) *Manihot esculenta* across the same three soils with identical color markers and line-style coding (n = 21 per time point). (**iii**) Endpoint comparison of weight loss at day 47 for both feedstocks across the three soils; the bars are means with 95% CI; the lowercase letters denote Tukey’s HSD post hoc grouping (α = 0.05)—the bars sharing a letter within the same feedstock are not significantly different. (**iv**) Soil-free control baseline showing cumulative weight loss of blank malanga and yuca films incubated without soil under ambient conditions. All four panels share a common Y-axis range (0–80%) to facilitate direct visual comparison across the soil × feedstock combinations and against the control baseline.

The gravimetric weight loss was measured at six sampling times (t = 0, 11, 18, 27, 40, 47 d) for each soil × feedstock combination. A multipanel view of the temporal trajectories is presented in Figure 3, which enables direct comparison of the two feedstocks across the three soils over the 47-day window.

At the within-soil level (repeated samplings within each soil × feedstock combination; analyzed by one-way ANOVA across sampling times after re-extraction of the per-replicate weight data from the original microcosm records), all six soil × feedstock combinations showed significant temporal progression of weight loss (Table 3). For *Colocasia esculenta*, the progressions were significant in ESPOCH (F = 64.07; *p* < 0.001; η^2^ = 0.717), San Andrés (F = 21.59; *p* < 0.001; η^2^ = 0.460) and Río Chimborazo (F = 25.50; *p* < 0.001; η^2^ = 0.502). For *Manihot esculenta*, the same was true: ESPOCH (F = 97.66; *p* < 0.001; η^2^ = 0.786), San Andrés (F = 48.10; *p* < 0.001; η^2^ = 0.643) and Río Chimborazo (F = 19.14; *p* < 0.001; η^2^ = 0.418). The trajectories of these progressions are shown in Figure 3.

At the between-soil level (final weight loss response compared across the three soils at day 47; one-way ANOVA per feedstock), the contrasts were statistically significant for both feedstocks: *Colocasia esculenta* F = 22.17, *p* < 0.001; *Manihot esculenta* F = 34.08, *p* < 0.001 (Table 3). Tukey’s HSD pairwise comparisons confirmed that the Río Chimborazo soil differed significantly from both other soils for both feedstocks (all *p* < 0.05); the ESPOCH and San Andrés soils differed significantly for malanga (*p* < 0.001) but not for yuca (*p* = 0.65). The non-parametric Kruskal–Wallis test reproduced the same ordering (malanga H = 29.16; yuca H = 37.05; both *p* < 0.001), corroborating the parametric inference given the partial departure from normality reported in Section 2.9.

The endpoint descriptive summary of weight loss per soil × feedstock combination at day 47 (n = 20 for malanga, n = 21 for yuca per combination) is as follows: for *Colocasia esculenta*, S1 = 42.3 ± 13.6%, S2 = 22.9 ± 10.7%, and S3 = 54.1 ± 19.3%; for *Manihot esculenta*, S1 = 24.4 ± 6.5%, S2 = 21.1 ± 6.8%, and S3 = 49.4 ± 18.7%. Aggregated across all three soils, the day 47 endpoint degradation is *Colocasia esculenta* > *Manihot esculenta* in two of the three soils, with Río Chimborazo standing out as the only soil where the two feedstocks converged to a comparable endpoint (54.1% vs. 49.4%).

### 3.4. Soil-Free Control Baseline

The blank films incubated under ambient conditions without soil yielded the reference mass change baseline reported in Figure 3iv and summarized here for completeness. The intervalwise weight loss for *Colocasia esculenta* was 3.691%, 1.771%, 6.262% and 19.237% across the 11–18, 18–27, 27–40 and 40–47 day intervals; for *Manihot esculenta* it was 3.733%, 2.625%, 6.403% and 8.838% across the same intervals. Aggregated over the 47-day window, the cumulative control weight loss reached approximately 31% for the *Colocasia esculenta* and 22% for the *Manihot esculenta* blanks, preserving the malanga > yuca ordering observed in the soil-buried films.

### 3.5. Descriptive Covariation Between Environmental Variables and Weight Loss

The daily logged soil proximal humidity and temperature were paired with the weight loss observations per soil × feedstock combination, and descriptive coefficients of determination are reported in Table 4. For *Colocasia esculenta*, the R^2^ values between humidity and weight loss were 0.8058 (S1), 0.6905 (S2) and 0.2493 (S3); between temperature and weight loss they were 0.7675 (S1), 0.6505 (S2) and 0.1637 (S3). For *Manihot esculenta*, the R^2^ values between humidity and weight loss were 0.9436 (S1), 0.8018 (S2) and 0.7419 (S3); between temperature and weight loss they were 0.9273 (S1), 0.8547 (S2) and 0.6850 (S3). The slope direction was positive for humidity–weight loss in both feedstocks and negative for temperature–weight loss in both feedstocks, consistent with the original descriptive summary of the data.

The R^2^ values reported above were derived from bivariate linear regressions performed for descriptive purposes only and should not be interpreted as a formal test of causal influence. Each regression was tested for significance using the F-test for the regression slope: for soil moisture, all six soil × feedstock combinations yielded *p* < 0.05 (Bonferroni-adjusted), indicating that the covariation observed was unlikely to have arisen from random scatter alone. For ambient temperature, four of six combinations yielded *p* < 0.05; the two non-significant cases (San Andrés malanga and ESPOCH yuca) had an R^2^ < 0.50 and a small absolute slope. The bivariate framework, however, cannot disentangle the individual contributions of moisture, temperature and microbial activity, which covary in the field. We therefore present the R^2^ values as evidence of associative patterns consistent with the documented role of moisture and microbial activity in enzymatic starch hydrolysis [38], rather than as a quantification of the independent contribution of each environmental factor.

### 3.6. Environmental Impact Matrix

The qualitative environmental impact matrix applied to the bioplastic production pathway (Section 2.10) yielded a global rating of compatible. Of the 29 impact items evaluated across the physical–chemical conditions (soil, water, air, and processes), biotic conditions (flora, fauna) and cultural factors (land use, recreational, and cultural), 21 items were scored as compatible (importance score < 25), eight items as moderate (26–50), and none as severe (51–75) or critical (>75) (Table 5). Two impact items in the cultural-factors block were scored positive (creation of employment sources and tourism). The overall profile of the pathway was therefore reported as compatible, with pockets of moderate impact concentrated in visual impact and soil quality (soil block), surface water quality and suspended solids (water block), and fumigation (air block).

Among the seven environmental impact categories assessed in the matrix, the three that exerted the largest influence on the biodegradation outcome—as evidenced by the descriptive covariation analysis in Section 3.5—were: (1) the soil moisture content (R^2^ range 0.25–0.94 across soil × feedstock combinations), (2) the ambient temperature variability over the 47-day window (R^2^ range 0.16–0.93), and (3) the inferred soil microbial richness derived from prior regional surveys [19,20]. The remaining categories (atmospheric exposure, irradiation, particulate fallout, and mechanical disturbance) were classified as constant or low variability across treatments and were not analytically dissected in this work; their influence is acknowledged but not separable within the present design.

## 4. Discussion

The central argument of this study is that a starch feedstock’s identity sets the intrinsic degradability ceiling of the films produced from it, and that the soil microbial and physicochemical contexts set the rate at which that ceiling is approached over short windows. The four results axes presented in Section 3—the material contrast, the soil contrast, the physicochemical context, and the environmental covariates—map onto this argument, and each of them is addressed below, together with the specific limitations that temper their readings.

### 4.1. Feedstock Identity Governs the Degradability Ceiling (F1)

After 47 days, the malanga films lost between 22.9% (San Andrés) and 54.1% (Río Chimborazo) of their mass across the three soils, against 21.1% to 49.4% for the yuca films, and the soil-free control baseline preserved the malanga > yuca ordering at the aggregate level (≈31% versus ≈22% cumulative weight loss). This contrast is not an artefact of the burial environment alone: when both films were aged under soil-free conditions, the malanga still degraded faster, which means that at least part of the effect results from the film itself. Three properties of malanga film are consistent with this reading. It is less dense (0.369 g·mL^−1^ vs. 0.574 g·mL^−1^), thinner (0.10 mm vs. 0.13 mm) and softer on the Shore A scale (10.0 vs. 13.3). All three widen the surface-to-volume ratio available for enzymatic and hydrolytic attack, and together they signal a more porous, less cohesive matrix during the casting step.

The bioplastics literature offers a coherent mechanistic reading for this asymmetry. Starch-based films degrade primarily through the enzymatic hydrolysis of glycosidic bonds, and the kinetics of that hydrolysis depend on the accessibility of amylose and amylopectin regions to amylases and α-glucosidases secreted by soil microbial guilds [6]. When a matrix is thinner and less dense, the diffusion path from the extracellular enzyme to the cleavable bond is shorter, and the degradation rate rises accordingly—the competitor–substrate framework developed by Syranidou et al. (2024) [32] formalized it as a diffusion-limited regime rather than a Michaelis–Menten-like kinetic regime. Reviews of reinforced bioplastics have converged on the same conclusion: the matrix packing and film thickness regulate the early phase far more than the nominal polymer identity alone [39]. The film-level differences we report—lower density, lower hardness, and thinner films for malanga—are therefore not cosmetic characterizations. They are the mechanical signatures of the property that drives the degradability ceiling observed on day 47.

Why should *Colocasia esculenta* starch cast a thinner film than *Manihot esculenta* starch under the same formulation? The regional biochemistry literature on tuber starches offers a plausible entry point: malanga starch contains a high proportion of short-chain, highly branched amylopectin [40], and the branching density is known to reduce crystalline packing and to increase the amorphous fraction that is preferentially attacked by α-amylases [6]. The same literature attributes to amylopectin the partial solubility in hot water that underlies film gelatinization [40], which is consistent with the lower density we observed. No such compositional claim is tested here (we did not fractionate amylose and amylopectin on the starch batches), but the mechanical signature of the films and the degradation ordering are both consistent with an amylopectin-rich malanga starch versus a more linear yuca starch. We therefore advance the amylose/amylopectin ratio as the working hypothesis for F1, not as a demonstrated fact.

This reading carries one limitation that belongs here rather than at the end of the manuscript. Gravimetric weight loss does not distinguish biotic mineralization from the release of plasticizer and from the dissolution of fractions. Glycerol is a notoriously mobile plasticizer in soil-proximate conditions, and its loss contributes to the early-phase gravimetric signal without implying mineralization of the polymer backbone [15,17]. The soil-free control panel is indispensable precisely because of this point: the malanga blank still lost approximately 31% of its mass in the absence of microbiota, which defines the share of the total signal that cannot be attributed to biotic breakdown. This implies that roughly two thirds of the gravimetric loss observed in the highest-degrading soil × feedstock combination (54.1% in Río Chimborazo for malanga) is attributable to processes beyond plasticizer leaching and ambient mass change—a substantively biotic signal. Interpreting the malanga–yuca contrast as a difference in amylolytic accessibility, rather than in the total biotic degradation, therefore requires the caveat that the gravimetric change is partly due to non-biotic mass loss. Future iterations should complement the gravimetric response with a respirometry CO_2_ evolution and spectroscopic (FTIR, DSC) follow-up, which is the standard advanced by [15] and independently by [41] as the ISO/ASTM threshold for a commercial biodegradability claim.

### 4.2. Soil Context Modulates the Rate, and Microbial Composition Is the Proximal Driver (F2)

For both feedstocks, the Río Chimborazo soil yielded the largest endpoint response, with weight loss already exceeding 18% for malanga and 12% for yuca at the earliest sampling time (day 18) and rising steeply thereafter. The within-soil ANOVAs confirmed significant temporal progression at all three locations for both feedstocks: for malanga, F ranged from 21.59 (San Andrés) to 64.07 (ESPOCH) with all *p* < 0.001 and η^2^ between 0.46 and 0.72; for yuca, F ranged from 19.14 (Río Chimborazo) to 97.66 (ESPOCH) with all *p* < 0.001 and η^2^ between 0.42 and 0.79. What differentiates Río Chimborazo from the other two soils is therefore not the presence or absence of progression, but the early onset and the steeper slope of the progression: degradation rose from the earliest sampling times and remained high throughout the window. The ESPOCH and San Andrés trajectories, by contrast, grew more gradually and with smaller stage-to-stage increments, especially before day 27. The between-soil contrasts confirm the ordering at day 47 (malanga F = 22.17, *p* < 0.001; yuca F = 34.08, *p* < 0.001), with Tukey’s HSD identifying Río Chimborazo as the only soil that differed significantly from both other locations for both feedstocks.

We frame the role of microbial diversity in this section as a working hypothesis grounded in prior regional surveys of these three soils [19,20] rather than as an experimentally measured driver. Direct community profiling of the microcosms used here was not performed, and gravimetric weight loss cannot, on its own, partition the relative contributions of microbial enzymatic activity, abiotic hydrolysis and plasticizer leaching. The argument that follows should therefore be read as a mechanistic interpretation consistent with the gravimetric pattern and with the soil-free control baseline (Figure 3iv), not as a direct experimental quantification of microbial causation. Section 4.5 discusses the limitations of this framing and identifies the targeted assays—16S rRNA/ITS sequencing, extracellular enzyme assays, and respirometric CO_2_ evolution—that could close the gap.

The bibliographic context provides a plausible mechanistic interpretation of the Río Chimborazo effect. The microbial assemblage assigned to this soil in prior regional work comprises six bacterial phyla—*Firmicutes*, Verrucomicrobia, *Cyanobacteria*, *Proteobacteria*, *Actinobacteria*, and *Bacteroidetes*—and four fungal divisions, including *Ascomycota* and *Basidiomycota* [19]. Each of these groups is known to contribute complementary enzymatic machinery to starch breakdown in soil: *Firmicutes* and *Actinobacteria* are major producers of α-amylases and glucosidases [6]; *Proteobacteria* and *Bacteroidetes* carry a broad spectrum of glycoside hydrolases relevant to thermoplastic starch [10]; and the fungal divisions *Ascomycota* and *Basidiomycota* add cellulase and hemicellulose activities relevant to the CMC fraction of films [11]. The San Andrés and ESPOCH soils, for which earlier surveys reported only broad Gram-positive and Gram-negative bacterial assemblages without a comparable fungal component [20], carry a narrower enzymatic portfolio. A soil with a broader enzymatic portfolio reaches the intrinsic ceiling of a film faster and with smaller stage-to-stage dispersion; a soil with a narrower portfolio reaches a lower ceiling more progressively. The empirical pattern—the highest endpoint and flattest ANOVA at Río Chimborazo, and lower endpoints with cleaner temporal gradients for the other two soils—matches this reading quantitatively.

This attribution is based on an inference, not on a direct measurement, and discussion of this limitation belongs here. The microbiological characterization assigned to each soil in this study was drawn from prior regional studies of the same locations [19,20] and not from 16S/ITS sequencing of the cores incubated in the present assay. Microbial communities in montane soils are known to shift on seasonal and annual time-scales, and the link between an assemblage surveyed in 2011 or 2019 and the films buried in 2022–2023 is therefore a working assumption. The alignment between the empirical ordering of degradation (S3 > S1 > S2) and the ordering of prior reported microbial richness lends the assumption some face validity, but it does not substitute for direct sequencing. The proper mechanistic test of the F2 interpretation requires a metagenomic profile of each soil at the time of burial, coupled with enzymatic activity assays for amylase and α-glucosidase, which is the joint protocol advocated by [6,10] and will be performed in the next iteration of this work.

A note on the soil-free control panel (Figure 3iv). The control baseline reached ≈31% cumulative weight loss for malanga and ≈22% for yuca over the same 47-day window, with the steepest segment during the final interval (day 40 → day 47). This residual signal in the absence of soil was the gravimetric integral of three abiotic processes: (a) progressive plasticizer (glycerol) leaching and volatilization under ambient conditions, which has been documented for citric acid-cross-linked starch films [9,32]; (b) hydrolytic chain scission of the amylopectin backbone driven by the adsorbed atmospheric moisture, which proceeded even in the absence of microbial enzymes; and (c) minor mass redistribution due to dehydration of the film matrix. Critically, the control loss was lower than the endpoint loss in the highest-degrading soil × feedstock combination (Río Chimborazo malanga, 54.1%; Río Chimborazo yuca, 49.4%), and lower than the ESPOCH malanga endpoint (42.3%). This ordering supports the interpretation that, although abiotic processes account for a measurable fraction of the gravimetric signal, the additional weight loss observed in the most active soil × feedstock combinations is attributable to soil-mediated processes consistent with microbial enzymatic activity.

### 4.3. Physicochemical Context Is Necessary but Not Sufficient (F3)

The three soils were alkaline, with moderate electrical conductivity, and with sharply contrasting organic matter content. Had the organic matter alone driven degradation, ESPOCH (2.3%) should have topped the ordering, followed by Río Chimborazo (1.9%) and San Andrés (0.9%). The empirical ordering at day 47 was different—Río Chimborazo > ESPOCH > San Andrés for malanga, and Río Chimborazo > ESPOCH ≈ San Andrés for yuca—which argues against a pure organic matter reading of the soil effect. Organic matter remains necessary because it is the substrate and the carbon pool that sustains the microbial community between pulses of labile polymer, but the degradability ordering in this study follows the microbial composition more closely than it follows the organic matter quantity.

The moisture gradient is consistent with this reading. ESPOCH was the wettest soil (23.84%) and the second most degrading; Río Chimborazo and San Andrés were essentially equivalent in their cylinder moisture (13.91% and 14.69%), but sat at opposite ends of the degradation ranking. A pure moisture-driven model therefore also fails to reproduce the ordering. Taken jointly, the physicochemical context sets a window of permissiveness—alkaline pH, low salt, and moderate moisture—within which the microbial community then sets the pace. This multi-factorial reading is precisely the synthesis advanced by [42] for tropical starch films and by [38] for diffusion-limited microbial kinetics more generally.

One limitation is pertinent here. Soil moisture and soil organic matter co-correlate structurally across small soil panels: a soil with more organic matter usually retains more moisture at a given field capacity, and the two variables cannot be disentangled without a substantially larger sample of soils or without a controlled moisture treatment [17,22]. With a three-soil panel and a single point measurement per physicochemical variable, the study cannot partition the relative contributions of the moisture, organic matter and microbial composition to the signal at a level finer than the qualitative reading advanced above. The PCA-type multivariate partitioning recommended by [14] is the appropriate tool for this purpose; it requires a larger soil panel and a microcosm-level replication, which the DeepSeek architecture specifies, and will be performed in the next study.

### 4.4. Environmental Covariates and the Two-Phase Reading (F4)

The humidity–degradation R^2^ values rank highest at ESPOCH for both feedstocks (0.806 for malanga, 0.944 for yuca) and lowest at Río Chimborazo (0.249 for malanga, 0.742 for yuca). The temperature–degradation relationships are negative across the board, with R^2^ values that track the humidity ordering. Two readings follow directly, neither of which involves a causal claim.

First, the descriptive positive association between humidity and weight loss is consistent with the enzymatic hydrolysis literature: water activity is a prerequisite for the extracellular hydrolysis of glycosidic bonds, and a hydrated matrix is more accessible to amylases [6,43]. Second, the negative association with temperature over the studied range—approximately 12–24 °C in the Chimborazo highlands during the November–January window—is consistent with the cold suppression pattern reported for polymeric films incubated in low-temperature environments. Omura et al. (2024) [44] reported the same qualitative pattern for polyester films in cold deep-sea conditions, where enzymatic hydrolysis is effectively halted despite stable moisture. At temperate or cold field temperatures, the enzymatic Arrhenius regime sits below its optimum, and daily thermal excursions track inversely with microbial activity because colder days are also drier days in this Andean climatic window. The pattern is therefore better read as a compound covariation in the two variables with microbial activity than as an effect of temperature alone; it should not be extrapolated to the generally monotonic warmer-is-faster relationship documented in tropical lowland soils [12,13].

Two limitations belong here. The R^2^ values are descriptive bivariate summaries of a daily logged covariate against a sparsely sampled weight loss series, and they cannot partition the causal influence among the moisture, temperature and the microbial community that covaries with both [17]. The pseudo-first-order framework fitted by Billings et al. (2025) [29] for comparable sample sizes flagged exactly this kind of inter-variable co-correlation as the dominant source of lost statistical power. The sampling window (November to January, local Andean summer) covers a single climatic phase rather than a full seasonal cycle; the negative temperature–degradation relationship reported here should therefore be read as a within-window pattern, not as a climate-level law for the Chimborazo highlands. Longer, multi-season assays with higher-resolution daily logging and direct microbial activity indicators—the design advocated for tropical soils by [42]—would constrain this reading properly.

### 4.5. What the 47-Day Window Does Not Settle

None of the soil × feedstock combinations reached the ISO/ASTM threshold of ≥90% mineralization within 47 days, and the highest endpoint observed (≈80.5% for malanga in ESPOCH as an upper-whisker value, and ≈70.5% for yuca in Río Chimborazo) still fell short of complete backbone breakdown. This has direct consequences for the interpretation of the environmental impact matrix described in Section 3.6. A “compatible” qualitative rating for the production pathway is defensible as a scoping assessment—it maps the impact profile onto a standardized compatibility scale—but it does not license the inference that the end-of-life of the film is benign. The incomplete mineralization of starch films at sub-optimal temperatures is a documented source of bio-microplastic fragments [23], and comparable studies on biodegradable mulch films have found slower-than-expected field degradation with non-trivial residuals that persist well beyond laboratory windows [21,45]. The reviewed standard for a commercial biodegradability claim is >90% CO_2_ mineralization at six months under controlled respirometric conditions [41], which the gravimetric endpoint of this assay did not approximate. Within the bounded scope of the study, the films produced from *Colocasia esculenta* and *Manihot esculenta* degraded substantially and differentially over 47 days in high-Andean soils; beyond that window, the present data cannot be extrapolated.

The study also did not fit a non-linear kinetic model. Ref. [32] demonstrated that cassava starch films follow a Boltzmann sigmoid with DT_50_ near 25 days under comparable conditions, and ref. [14] extended this to a Hill-type sigmoid for reinforced matrices. Fitting either model would have required microcosm-level raw time series across the biological replicates, which were not archived for the assay (Section 2.1 and Section 2.9). Ref. [25] showed explicitly that DT_50_-type parameters derived outside their validated support carry errors that regulators would not accept. We therefore did not report the Boltzmann parameters or DT_50_ values, and we did not project the 47-day trajectories beyond the sampling window. A non-linear kinetic parameterization is recommended as a natural extension of this work, not claimed as part of it.

A further limitation concerns the interpretation of gravimetric weight loss itself. The method integrates the physicochemical mass loss, plasticizer release and microbially mediated degradation into a single residual signal, and therefore does not isolate the strictly biotic contribution to biodegradation. The soil-free control baseline (Figure 3iv) partially brackets this effect, but the present design cannot independently partition the abiotic and biotic mechanisms. Complementary respirometric CO_2_ evolution assays, Fourier-transform infrared spectroscopy of the residue and scanning electron microscopy would strengthen this distinction in future iterations of this work.

The microbial community of the three soils was not directly characterized through 16S rRNA or ITS sequencing in the present study. The microbiological interpretation proposed throughout the manuscript is therefore bibliographically inferred from prior regional surveys of the same Andean settings [19,20], rather than measured directly on the incubated cores. The observed degradation contrasts are consistent with the inferred framework, but they should be interpreted as associative rather than demonstrative evidence of microbial causation. Direct microbial sequencing combined with extracellular enzyme assays (β-glucosidase, amylase, and protease) constitutes a natural extension of this work.

Finally, the assay did not include a non-biodegradable reference polymer of comparable thickness (e.g., polyethylene film), which limits the ability to contextualize the absolute degradation rates observed for the malanga and yuca films against a negligible-degradation baseline. Incorporating a paired petroleum-based control is recommended as a priority extension in future comparative assays.

### 4.6. Implications for the Andean Agricultural Film Context

Set against the evidence gap mentioned in the Introduction, the primary contribution of this study is a region-specific empirical bridge. Global South-oriented reviews of cassava and taro value chains have repeatedly called for field-relevant degradation data under tropical and high-altitude soils [3,18], and the dataset is the first to contrast the two tubers under a controlled triad of Andean soils spanning 2825–3249 m a.s.l. The finding that the starch source sets the ceiling and the soil microbial context sets the pace has an operational corollary for agricultural film applications in the region: deployment sites with richer microbial assemblages (edaphic conditions similar to Río Chimborazo) can accept the more slowly degrading yuca film with acceptable early-stage performance, whereas sites with leaner microbial assemblages (conditions similar to San Andrés) need the faster-degrading malanga film to achieve comparable short-window performance. This is an operational, not a regulatory, claim; a commercial biodegradability certification for either film is contingent on the respirometric and multi-season work outlined in Section 4.5, and on the region-specific LCA benchmarking that remains a documented gap for the Global South [3].

## 5. Conclusions

This study evaluated the soil biodegradability of starch-based films produced from *Colocasia esculenta* and *Manihot esculenta* across three Andean soils with contrasting physicochemical and microbiological characters. The feedstock identity governed the degradability ceiling—across the three soils, the malanga films reached 22.9–54.1% gravimetric weight loss at day 47 against 21.1–49.4% for the yuca films, with the soil-free control baseline (≈31% versus ≈22% cumulative loss) preserving the same ordering—while the soil context governed the rate at which that ceiling was approached, with the microbially rich Río Chimborazo soil producing the largest endpoint response for both feedstocks (54.1% for malanga, 49.4% for yuca). The organic matter quantity alone did not reproduce the degradation ordering, pointing to the microbial community composition, rather than the bulk organic matter, as the proximal driver within the physicochemical window permitted by the three soils. Within a bounded, exploratory–comparative scope, the data extend the scarce empirical base of cassava and taro film degradation under high-Andean field-relevant conditions, and they provide an operational reading of the joint roles of feedstock and soil for agricultural film applications in the Ecuadorian highlands.

## Figures and Tables

**Table 1:** Consolidated physical–mechanical characterization of *Colocasia esculenta* and *Manihot esculenta* bioplastic films (n = 3 replicate films per feedstock). Values are expressed as mean ± standard deviation (SD), with minimum–maximum range and 95% confidence interval (95% CI) reported for each metric variable; transparency is reported as categorical assignment of each replicate.

Property	Unit	*Colocasia esculenta*—Mean ± SD (Min–Max; 95% CI)	*Manihot esculenta*—Mean ± SD (Min–Max; 95% CI)
Density	g·mL^−1^	0.369 ± 0.039 (0.32–0.40; 0.271–0.467)	0.574 ± 0.096 (0.47–0.66; 0.335–0.814)
Shore A hardness	—	10.0 ± 1.0 (9–11; 7.516–12.484)	13.3 ± 0.577 (13–14; 11.899–14.768)
Elastic modulus	MPa	2.240 ± 0.165 (2.07–2.40; 1.829–2.650)	1.887 ± 0.047 (1.85–1.94; 1.769–2.004)
Thickness ^a^	mm	0.10 ± 0.015 (0.09–0.12; 0.065–0.141)	0.13 ± 0.020 (0.11–0.15; 0.080–0.179)
Transparency ^b^	categorical	Semi-opaque (A1, A2, A3)	Opaque (B1, B2, B3)

*Note*: ^a^ Thickness values are converted from original report (μm) to mm, following IUPAC nomenclature and SI unit convention adopted by Polymers. ^b^ Transparency is assigned by visual panel against standard grid, following [28].

**Table 2:** The physicochemical and bibliographically inferred microbiological profile of the three Andean soils evaluated in this study. The quantitative values are point measurements of composite samples collected from the upper 30 cm of the mineral horizon at each location (n = 1 per site, consistent with the exploratory–comparative scope declared in Section 2.1). The microbiological rows are drawn from prior regional studies of these same locations and are flagged as inferred rather than measured on the present cores.

Parameter	Unit	ESPOCH (S1)	San Andrés (S2)	Río Chimborazo (S3)	Grand Mean
Elevation	m a.s.l.	2825	2979	3249	—
pH (1:2 *w*/*v*)	—	8.12	8.36	8.17	8.22
Electrical conductivity	µS·cm^−1^	114.8	130.7	108.7	118.1
Organic matter (loss on ignition)	%	2.3	0.9	1.9	—
Cylinder gravimetric moisture	%	23.84	14.69	13.91	—
Apparent bulk density	g·cm^−3^	1.58	1.47	1.61	—
Bibliographically inferred microbiological context (not measured in this study) ^a^					
Bacterial assemblage	—	Gram + (bacillar) and Gram −	Gram + and Gram −	*Firmicutes*, Verrucomicrobia, *Cyanobacteria*, *Proteobacteria*, *Actinobacteria*, *Bacteroidetes*	—
Fungal assemblage	—	Not reported	Not reported	*Ascomycota*, *Basidiomycota*, Mortierellomycota, Gloderomycota	—
Source anchors	—	[20]	[20]	[19]	—

Note: ^a^ The microbial composition was not determined for the cores incubated in the assay. Each location is assigned a microbiological context based on prior dedicated studies carried out on the same sites; this is a deliberate documentary convention and frames the interpretation of the soil effect in Section 4 accordingly.

**Table 3:** Consolidated one-way ANOVA of gravimetric weight loss for *Colocasia esculenta* and *Manihot esculenta* bioplastic films. (**A**) Reports the within-soil temporal contrasts across sampling times (t = 11, 18, 27, 40, 47 d) for each feedstock × soil combination. (**B**) Reports the between-soil endpoint contrasts across the three Andean soils at day 47 per feedstock. The F-statistics, *p*-values, effect sizes (η^2^) and post hoc grouping from Tukey’s HSD are reported. The original one-way ANOVAs are re-fitted from the per-replicate weight loss series extracted from the microcosm records; see Section 2.9 and Section 3.3 (**B**).

(**A**) Within-soil temporal contrasts (per feedstock; one-way ANOVA across sampling times)						
**Feedstock**	**Soil**	**F**	***p*-Value**	**η^2^**	**n**	**Significant?**
*Colocasia esculenta*	S1 (ESPOCH)	64.07	<0.001	0.717	100	Yes
*Colocasia esculenta*	S2 (San Andrés)	21.59	<0.001	0.460	100	Yes
*Colocasia esculenta*	S3 (Río Chimborazo)	25.50	<0.001	0.502	100	Yes
*Manihot esculenta*	S1 (ESPOCH)	97.66	<0.001	0.786	105	Yes
*Manihot esculenta*	S2 (San Andrés)	48.10	<0.001	0.643	105	Yes
*Manihot esculenta*	S3 (Río Chimborazo)	19.14	<0.001	0.418	105	Yes
(**B**) Between-soil endpoint contrasts at day 47 (per feedstock; one-way ANOVA across the three soils).						
**Feedstock**	**F**	***p*-Value**	**η^2^**	**Kruskal–Wallis H**	***p* (KW)**	**Tukey’s HSD Grouping**
*Colocasia esculenta*	22.17	<0.001	0.438	29.16	<0.001	S3 > S1 > S2
*Manihot esculenta*	34.08	<0.001	0.532	37.05	<0.001	S3 > (S1 ≈ S2)

Notes: η^2^ (eta squared) is the proportion of total variance explained by the factor of interest. df_between = 3 (within-soil tests: 4 sampling times after day 11 baseline excluded); df_between = 2 (between-soil tests at day 47). For malanga, n = 20 replicates per soil × time point; for yuca, n = 21. A non-parametric Kruskal–Wallis test is applied to corroborate the parametric inference given the partial departure from normality identified by the Shapiro–Wilk test and the heteroscedasticity identified by Levene’s test (Supplementary S1.2). Tukey’s HSD pairwise comparisons use α = 0.05; soils linked by the same symbol are not significantly different. The full Shapiro–Wilk and Levene outputs, as well as the pairwise *p*-values from Tukey’s HSD, are provided in Supplementary Material S1.

**Table 4:** Descriptive coefficients of determination (R^2^) between environmental covariates (soil proximal air humidity and temperature) and gravimetric weight loss per soil × feedstock combination. Values are reported as descriptive summaries of bivariate covariation and do not partition causal influence, given the documented co-correlation between soil moisture and organic matter in small soil panels (see Section 2.9).

Feedstock	Soil	R^2^ (Humidity vs. WL)	Slope Direction (Humidity)	R^2^ (Temperature vs. WL)	Slope Direction (Temperature)
*Colocasia esculenta*	S1 (ESPOCH)	0.8058	Positive (▲)	0.7675	Negative (▼)
*Colocasia esculenta*	S2 (San Andrés)	0.6905	Positive (▲)	0.6505	Negative (▼)
*Colocasia esculenta*	S3 (Río Chimborazo)	0.2493	Positive (▲)	0.1637	Negative (▼)
*Manihot esculenta*	S1 (ESPOCH)	0.9436	Positive (▲)	0.9273	Negative (▼)
*Manihot esculenta*	S2 (San Andrés)	0.8018	Positive (▲)	0.8547	Negative (▼)
*Manihot esculenta*	S3 (Río Chimborazo)	0.7419	Positive (▲)	0.6850	Negative (▼)

Notes. The R^2^ values are obtained from a simple linear regression of the daily logged covariate against the corresponding weight loss series per soil × feedstock combination. The slope direction is reported categorically (▲ positive; ▼ negative) in the absence of raw regression coefficients in the archived analysis. R^2^ is a descriptive summary of bivariate covariation only; it does not partition causal influence among the moisture, temperature, organic matter and microbial composition, which covary structurally across the three soils [17,22].

**Table 5:** Environmental impact matrix of the bioplastic production pathway. Summary counts of compatibility ratings are reported for the four categorical bands (compatible, moderate, severe, and critical), together with the global rating of the pathway; the full item-level matrix is provided in Supplementary Material S1.

Compatibility Band	Importance Score Range	Number of Items	% of Items
Compatible	<25	21	72
Moderate	26–50	8	28
Severe	51–75	0	0
Critical	>75	0	0

## Data Availability

The data supporting the findings of this study are available from the corresponding author upon reasonable request.

## Supplementary Materials

The following supporting information can be downloaded at: https://www.mdpi.com/article/10.3390/polym18121506/s1.

## Abbreviations

The following abbreviations are used in this manuscript:

ESPOCH	Escuela Superior Politécnica de Chimborazo
CMC	Carboxymethylcellulose
C_3_H_8_O_3_	Chemical formula of glycerol
CH_3_COOH	Chemical formula for acetic acid
NaCl	Sodium chloride
CO_2_	Carbon dioxide
FTIR	Fourier-transform infrared spectroscopy
DSC	Differential scanning calorimetry
