# Supplementary material for: Soil Physicochemical Parameters and Bibliographically Inferred Microbial Diversity as Drivers of Early-Stage Biodegradation of Colocasia esculenta and Manihot esculenta Starch Bioplastics in Three High-Andean Soils of Ecuador
Source: Polymers (Basel). 2026 Jun 16;18(12):1506. doi: 10.3390/polym18121506 (PMC13307041; doi:10.3390/polym18121506)
Supplement: Supplementary file 1 [file polymers-18-01506-s001.zip › polymers-4297593-supplementary.pdf]

Supplementary Material S1

Detailed statistical output for the biodegradation assay

Manuscript: *Soil physicochemical parameters and bibliographically inferred microbial diversity as drivers of early-stage biodegradation of Colocasia esculenta and Manihot esculenta starch bioplastics in three high-Andean soils of Ecuador*

Journal: Polymers (MDPI) | Manuscript ID: polymers-4297593

Authors: María Núñez, Giorgina Carmilema, María Arias, David Puyol

This Supplementary Material reports the full statistical output that supports the inferential analysis presented in §3.3 and §4.2 of the main manuscript. The contents are organized into six sections:

S1.1 Shapiro–Wilk normality tests for all soil × feedstock × sampling-time groups.

S1.2 Levene's test for homogeneity of variances across soils.

S1.3 Tukey HSD pairwise post-hoc comparisons among soils at day 47.

S1.4 Kruskal–Wallis non-parametric verification.

S1.5 Descriptive statistics per soil × feedstock × sampling-time group.

S1.6 Methodological note on the statistical framework.

S1.1 — Shapiro–Wilk normality tests

Normality of the weight-loss distribution was tested independently for each soil × feedstock × sampling-time group (n = 20 per group for malanga; n = 21 per group for yuca). Day 0 and Day 11 are reported as baseline (all values = 0); the test was not informative at those time points. Significance level $\alpha = 0.05$; groups with $p < 0.05$ reject the null hypothesis of normality.

Table S1. Shapiro–Wilk normality test results per soil × feedstock × sampling-time group.

Feedstock	Soil	Day	n	W	p-value	Normal?
malanga	S1 ESPOCH	18	20	0.6934	< 0.001	No
malanga	S1 ESPOCH	27	20	0.8008	< 0.001	No
malanga	S1 ESPOCH	40	20	0.9134	0.074	Yes
malanga	S1 ESPOCH	47	20	0.8489	0.005	No
malanga	S2 San Andrés	18	20	0.9393	0.232	Yes
malanga	S2 San Andrés	27	20	0.7059	< 0.001	No
malanga	S2 San Andrés	40	20	0.6137	< 0.001	No
malanga	S2 San Andrés	47	20	0.8513	0.006	No
malanga	S3 Río Chimborazo	18	20	0.9816	0.953	Yes
malanga	S3 Río Chimborazo	27	20	0.8901	0.027	No
malanga	S3 Río Chimborazo	40	20	0.8794	0.017	No
malanga	S3 Río Chimborazo	47	20	0.9786	0.916	Yes
yuca	S1 ESPOCH	18	21	0.9779	0.892	Yes
yuca	S1 ESPOCH	27	21	0.9131	0.063	Yes
yuca	S1 ESPOCH	40	21	0.8513	0.005	No
yuca	S1 ESPOCH	47	21	0.9683	0.695	Yes
yuca	S2 San Andrés	18	21	0.9495	0.334	Yes
yuca	S2 San Andrés	27	21	0.9657	0.638	Yes
yuca	S2 San Andrés	40	21	0.9619	0.556	Yes
yuca	S2 San Andrés	47	21	0.9796	0.919	Yes
yuca	S3 Río Chimborazo	18	21	0.8285	0.002	No
yuca	S3 Río Chimborazo	27	21	0.7485	< 0.001	No
yuca	S3 Río Chimborazo	40	21	0.8237	0.002	No
yuca	S3 Río Chimborazo	47	21	0.8983	0.032	No

Summary: 13 of 24 non-baseline groups departed from normality. Departure is more frequent at S1 (ESPOCH) and S3 (Río Chimborazo) for malanga, and at S3 for yuca. The S2 (San Andrés) panel for yuca passed normality at all sampling times. The non-parametric Kruskal–Wallis test (Section S1.4) was therefore applied as a corroboration of the parametric inference.

S1.2 — Levene's test for homogeneity of variances

Levene's test was applied across the three soils, for each material × sampling-time combination, to test the homogeneity-of-variance assumption of the between-soil ANOVA. Significance level $\alpha = 0.05$; groups with $p < 0.05$ reject homogeneity.

Table S2. Levene's test results for homogeneity of variances across the three soils, per feedstock × sampling time.

Feedstock	Day	F	p-value	Homogeneous?
malanga	18	4.59	0.014	No
malanga	27	0.84	0.438	Yes
malanga	40	2.57	0.086	Yes
malanga	47	2.71	0.075	Yes
yuca	18	8.22	0.001	No
yuca	27	7.70	0.001	No
yuca	40	8.03	0.001	No
yuca	47	8.20	0.001	No

The yuca panel shows persistent heteroscedasticity across all sampling times, reflecting the very high variance of the Río Chimborazo response compared with the more compact distributions in ESPOCH and San Andrés. This is consistent with the substantially larger SD observed for yuca in S3 (18.73 % at day 47) relative to the other two soils (6.53 % and 6.79 %). The malanga panel shows heteroscedasticity only at day 18 and is homogeneous from day 27 onwards.

S1.3 — Tukey HSD pairwise comparisons at day 47

Post-hoc pairwise comparisons among the three soils were performed using the Tukey Honestly Significant Difference (HSD) procedure at $\alpha = 0.05$, separately for each feedstock.

Table S3. Tukey HSD pairwise comparisons among the three soils at day 47, per feedstock.

Feedstock	Comparison	Mean difference (%)	p-value	Significance
malanga	S1 ESPOCH vs S2 San Andrés	+19.40	< 0.001	***
malanga	S1 ESPOCH vs S3 Río Chimborazo	−11.86	0.040	*
malanga	S2 San Andrés vs S3 Río Chimborazo	−31.26	< 0.001	***
yuca	S1 ESPOCH vs S2 San Andrés	+3.30	0.652	ns
yuca	S1 ESPOCH vs S3 Río Chimborazo	−24.91	< 0.001	***
yuca	S2 San Andrés vs S3 Río Chimborazo	−28.21	< 0.001	***

Significance codes: *** $p < 0.001$; ** $p < 0.01$; * $p < 0.05$; ns $p \geq 0.05$. For malanga films, all three soils differ significantly from each other, with Río Chimborazo > ESPOCH > San Andrés. For yuca films, ESPOCH and San Andrés are statistically indistinguishable at day 47, while Río Chimborazo differs significantly from both.

S1.4 — Kruskal–Wallis non-parametric corroboration

Given that several soil × time groups departed from normality (Section S1.1) and that the yuca panel violated homogeneity of variances (Section S1.2), the between-soil endpoint contrasts at day 47 were corroborated with the non-parametric Kruskal–Wallis H test.

Table S4. Kruskal–Wallis non-parametric test on between-soil endpoint contrasts at day 47.

Feedstock	H statistic	df	p-value	Conclusion
malanga	29.16	2	< 0.001	Soils differ significantly
yuca	37.05	2	< 0.001	Soils differ significantly

The Kruskal–Wallis H test reproduces the inference of the parametric ANOVA: the three soils differ significantly in their endpoint biodegradation response for both feedstocks. This corroborates the parametric result and supports the interpretation advanced in §4.2 of the main manuscript.

S1.5 — Descriptive statistics per soil × feedstock × sampling time

Mean ± standard deviation (SD), with minimum and maximum, for each soil × feedstock × sampling-time group. Day 0 and Day 11 are reported as baseline (all values = 0).

Table S5. Descriptive statistics for gravimetric weight loss (%) per soil × feedstock × sampling-time group.

Feedstock	Soil	Day	n	Mean (%)	SD	Min	Max
malanga	S1 ESPOCH	18	20	7.56	6.12	1.14	30.46
malanga	S1 ESPOCH	27	20	8.44	6.53	1.35	30.67
malanga	S1 ESPOCH	40	20	22.01	7.86	10.89	41.54
malanga	S1 ESPOCH	47	20	42.25	13.63	27.41	85.65
malanga	S2 San Andrés	18	20	3.86	2.12	0.00	6.93
malanga	S2 San Andrés	27	20	7.03	9.84	−3.14	42.73
malanga	S2 San Andrés	40	20	14.75	7.19	8.15	42.83
malanga	S2 San Andrés	47	20	22.85	10.68	10.77	52.20
malanga	S3 Río Chimborazo	18	20	18.56	6.78	4.98	31.58
malanga	S3 Río Chimborazo	27	20	29.32	10.44	14.42	57.40
malanga	S3 Río Chimborazo	40	20	40.12	13.98	15.17	80.96
malanga	S3 Río Chimborazo	47	20	54.11	19.34	16.01	93.05
yuca	S1 ESPOCH	18	21	5.61	2.03	1.37	9.04
yuca	S1 ESPOCH	27	21	5.77	2.34	−0.17	11.66
yuca	S1 ESPOCH	40	21	14.99	4.12	1.61	20.18
yuca	S1 ESPOCH	47	21	24.44	6.53	13.60	36.21
yuca	S2 San Andrés	18	21	4.87	2.42	1.42	9.93
yuca	S2 San Andrés	27	21	5.80	4.27	−5.50	15.04
yuca	S2 San Andrés	40	21	12.44	5.34	3.51	26.47
yuca	S2 San Andrés	47	21	21.14	6.79	9.23	33.74
yuca	S3 Río Chimborazo	18	21	12.17	8.21	3.98	31.95
yuca	S3 Río Chimborazo	27	21	29.98	19.72	11.05	100.00
yuca	S3 Río Chimborazo	40	21	40.33	17.63	19.62	100.00
yuca	S3 Río Chimborazo	47	21	49.35	18.73	27.32	100.00

Note: *Negative minimum values for some groups (e.g. yuca S2 at day 27, −5.50 %) reflect minor positive net mass gain in a small fraction of replicates relative to baseline, attributable to residual soil adhesion despite the cleaning procedure (see §2.7 and §4.5 of the main manuscript). Maximum values of 100 % for yuca S3 at days 27–47 indicate complete disintegration of a subset of films. These observations are retained in the analysis as documented experimental records.*

S1.6 — Methodological note on the statistical framework

The analysis presented in §3.3 and §4.2 of the main manuscript was built around three deliberate methodological choices that warrant explicit acknowledgement here.

First, one-way ANOVA was applied within each soil to test temporal progression, and one-way ANOVA was applied across soils at day 47 to test the between-soil contrast. These tests assume independence of observations within groups. Because multiple films were incubated in shared containers (see §2.8 of the main manuscript), the design is pseudoreplicated in the sense of Hurlbert (1984) and the resulting F and p values are reported as descriptive summaries of the contrast under a nested grouping. They should not be interpreted as the output of an unconstrained factorial design with biologically independent replicates.

Second, a Linear Mixed Model (LMM) of the form weight_loss ~ time × soil × feedstock + (1 | microcosm) would be the appropriate statistical framework if microcosm-level identifiers were available for each film. The archived dataset reconstructs the per-replicate weight loss but does not preserve the microcosm-level grouping that would identify which films shared a container. Fitting an LMM without this information would impose an arbitrary grouping structure on the data; we therefore restrict the inference to the descriptive one-way ANOVA framework. The LMM analysis is recommended as the natural extension of this work in any follow-up study where microcosm-level metadata can be recorded prospectively (see §4.5 of the main manuscript).

Third, post-hoc adjustment for multiple comparisons in the within-soil tests was not applied beyond the implicit $\alpha = 0.05$ threshold of the individual ANOVAs. Given that all within-soil contrasts yielded $p < 0.001$ (Section A of Table 3 of the main manuscript), the inference is robust to any reasonable multiple-comparison correction (Bonferroni, Holm, or false-discovery rate). For the between-soil contrasts at day 47, Tukey HSD (Section S1.3 of this Supplementary Material) implements an integrated family-wise error rate control that is the standard in environmental and biological sciences.

All analyses were performed in Python 3.12 (scipy 1.16, numpy 2.0). The full per-replicate weight-loss dataset is available from the corresponding author on reasonable request.

End of Supplementary Material S1
